# Carfilzomib use in patients with relapsed/refractory multiple myeloma in France: A national retrospective cohort study

**DOI:** 10.1002/jha2.946

**Published:** 2024-07-23

**Authors:** Cyrille Hulin, Nadia Quignot, Heng Jiang, Hakima Mechiche, Gaëlle Désaméricq

**Affiliations:** ^1^ Hematology unit Hospital Center University De Bordeaux Bordeaux France; ^2^ Certara Evidence & Access Paris France; ^3^ Amgen SAS Boulogne‐Billancourt France

Carfilzomib is a proteasome inhibitor that has been shown to improve progression‐free survival and overall survival (OS) in patients with relapsed/refractory multiple myeloma (RRMM) [1, 2, 3]. In Europe, carfilzomib is approved for the treatment of patients with RRMM in combination with dexamethasone (Kd); lenalidomide and dexamethasone (KRd); and, since 2020, daratumumab and dexamethasone (D‐Kd) [4, 5, 6]. A recent observational cohort study described the use of KRd and Kd across Europe and Israel [7, 8], but survival data in the real‐world setting have not been reported for carfilzomib‐treated patients in Europe. Using and expanding on a previous study of patients with RRMM from the Système Nationale des Données de Santé (SNDS) national claims database [9], this comprehensive real‐world analysis describes the treatment patterns and outcomes of patients receiving carfilzomib (KRd and Kd) in France between 2016 and 2019.

Briefly, adults who were diagnosed with multiple myeloma and had received at least one dose of carfilzomib between 2014 and 2019 were included. The study design has previously been published [9]; this study extension had an end date of December 31, 2019. Carfilzomib became available in France in 2016 under an Authorization for Temporary Use, and then became fully available in July 2018. For each patient, data were collected on clinical characteristics and were analysed as a primary objective. Data on treatment patterns were also analysed. OS and time‐to‐next treatment (TTNT) were estimated in an exploratory analysis. OS was defined as the time from the start of carfilzomib treatment until death or the end of follow‐up. TTNT was defined as the start of carfilzomib treatment until the initiation of the next line of treatment or death.

The database included 2471 patients treated with carfilzomib‐based regimens. Clinical characteristics are presented by treatment group (KRd or Kd) and treatment line (second line [2L], third line [3L], and fourth or later lines [4L+]) in Table 1. Overall, 40% (*n* = 993) of patients received KRd. Half of patients (*n* = 497; 50%) receiving KRd initiated it as a 2L treatment, and 44%–60% had autologous stem cell transplantation (ASCT) at first line (1L). Most patients (*n* = 1478; 60%) received the Kd regimen, which was generally initiated as 4L+ (*n* = 1133; 77%). Among patients receiving Kd at 4L+, 40% had ASCT at 1L and most had previous exposure to bortezomib (98%), lenalidomide (91%), or daratumumab (60%) (Table 1).

**TABLE 1:** Patient characteristics.

	KRd (*n* = 993)			Kd (*n* = 1478)		
	2L	3L	4L+	2L	3L	4L+
Number of patients (*n*, %)	497 (50.1)	203 (20.4)	293 (29.5)	105 (7.1)	240 (16.2)	1133 (76.7)
Age at time of MM diagnosis (years)						
Median (range)	62 (27–96)	58 (18–84)	61 (18–80)	68 (27–84)	65 (34–86)	62 (22–87)
Male gender (*n*, %)	293 (59.0)	102 (50.2)	153 (52.2)	62 (59.0)	136 (56.7)	622 (54.9)
Comorbidities (*n*, %)^a^						
Congestive heart failure	≤10	≤10	≤10	≤10	≤10	52 (4.6)
Hypertension	61 (12.3)	33 (16.3)	56 (19.1)	27 (25.7)	45 (18.8)	253 (22.3)
Dementia	23 (4.6)	11 (5.4)	15 (5.1)	≤10	18 (7.5)	100 (8.8)
Chronic pulmonary disease	≤10	≤10	11 (3.8)	≤10	17 (7.1)	58 (5.1)
Diabetes	58 (11.7)	18 (8.9)	37 (12.6)	15 (14.3)	29 (12.1)	167 (14.7)
Moderate‐to‐severe renal disease	24 (4.8)	≤10	27 (9.2)	≤10	24 (10.0)	116 (10.2)
Any tumor^b^	25 (5.0)	≤10	22 (7.5)	≤10	21 (8.8)	97 (8.6)
Metastatic solid tumor	24 (4.8)	13 (6.4)	30 (10.2)	14 (13.3)	16 (6.7)	136 (12.0)
ASCT^c^ at 1L (*n*, %)	297 (59.8)	114 (56.2)	128 (43.7)	19 (18.1)	81 (33.8)	458 (40.4)
Radiotherapy before treatment initiation (*n*, %)	76 (15.3)	39 (19.2)	67 (22.9)	19 (18.1)	53 (22.1)	308 (27.2)
Previous treatments (*n*, %)						
Proteasome inhibitor based						
Carfilzomib based	≤10	≤10	27 (9.2)	≤10	≤10	159 (14.0)
Bortezomib based	471 (94.8)	199 (98.0)	288 (98.3)	77 (73.3)	218 (90.8)	1106 (97.6)
Bortezomib doublet/alone	168 (33.8)	77 (37.9)	206 (70.3)	42 (40.0)	137 (57.1)	813 (71.8)
Thalidomide/bortezomib	282 (56.7)	108 (53.2)	109 (37.2)	19 (18.1)	66 (27.5)	361 (31.9)
Daratumumab/bortezomib	≤10	≤10	40 (13.7)	≤10	≤10	132 (11.7)
IMiD based						
Pomalidomide based	≤10	≤10	210 (71.7)	≤10	29 (12.1)	827 (73.0)
Lenalidomide based	28 (5.6)	115 (56.7)	252 (86.0)	32 (30.5)	196 (81.7)	1032 (91.1)
Lenalidomide doublet/alone	≤10	84 (41.4)	191 (65.2)	17 (16.2)	151 (62.9)	750 (66.2)
Bortezomib/lenalidomide	18 (3.6)	29 (14.3)	81 (27.6)	15 (14.3)	37 (15.4)	226 (19.9)
Carfilzomib/lenalidomide	≤10	≤10	≤10	≤10	≤10	117 (10.3)
Daratumumab/lenalidomide	≤10	≤10	≤10	≤10	≤10	86 (7.6)
Ixazomib/lenalidomide	≤10	≤10	≤10	≤10	≤10	65 (5.7)
Daratumumab based	≤10	≤10	121 (41.3)	≤10	30 (12.5)	676 (59.7)
Other regimen with drugs of interest	≤10	≤10	94 (32.1)	≤10	24 (10.0)	543 (47.9)
Other unspecified chemotherapy	16 (3.2)	82 (40.4)	154 (52.6)	≤10	26 (10.8)	635 (56.0)
Number of deaths (*n*, %)	117 (23.5)	52 (25.6)	143 (48.8)	48 (45.7)	100 (41.7)	610 (53.8)
Number of patients with censor (*n*, %)	380 (76.5)	151 (74.4)	150 (51.2)	57 (54.3)	140 (58.3)	523 (46.2)
Mean (SD) follow‐up time from treatment initiation (months)	14 (11)	15 (12)	11 (9)	8 (8)	9 (8)	7 (8)

*Note*: Patient numbers of ≤10 are not specified to comply with data privacy restrictions in France.

Abbreviations: 1L, first‐line treatment; 2L, second‐line treatment; 3L, third‐line treatment; 4L+, fourth‐line treatment or later lines; ASCT, autologous stem cell transplantation; IMiD, immunomodulatory imide drug; Kd, carfilzomib and dexamethasone; KRd, carfilzomib, lenalidomide, and dexamethasone; MM, multiple myeloma; SD, standard deviation.

^a^ Comorbidities with ≥5% prevalence in any subgroup.

^b^ Including lymphoma and leukemia except for malignant neoplasm of skin.

^c^ Between 1L and 2L treatment initiation.

Patients receiving KRd were followed up for a mean of 11–15 months. The median OS estimates for patients receiving KRd were 40, 39, and 16 months at 2L (*n* = 497), 3L (*n* = 203), and 4L+ (*n* = 293), respectively. For patients initiating KRd in 2018, the median TTNT was longer when carfilzomib was received at earlier lines than later lines (2L, 14 months; 3L, 11 months; 4L+, 5 months) (Figure 1). For patients initiating KRd in 2019, the median TTNT was not reached at 2L and 3L and was 8 months in 4L+.

**FIGURE 1:**
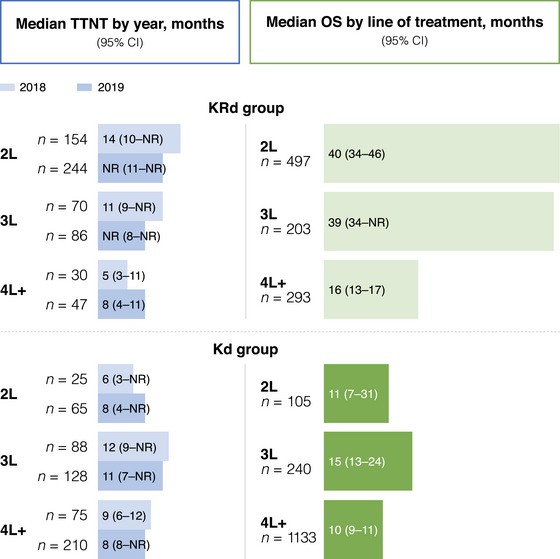
Median TTNT in 2018 and 2019 and median OS over the study period. OS included all patients treated with carfilzomib between 2016 and 2019. 2L, second‐line treatment; 3L, third‐line treatment; 4L+, fourth‐line treatment or later lines; CI, confidence interval; Kd, carfilzomib and dexamethasone; KRd, carfilzomib, lenalidomide, and dexamethasone; NR, not reached; OS, overall survival; TTNT, time‐to‐next treatment.

Patients receiving Kd were followed up for a mean of 7–9 months. The median OS estimates for patients receiving Kd were 11, 15, and 10 months at 2L, 3L, and 4L+, respectively. The median TTNT for Kd was similar between 2018 and 2019 by treatment line, but was longer for patients in 3L in both years at 12 and 11 months, respectively (Figure 1).

The median concentration of the first two doses of carfilzomib was consistent across the treatment groups (KRd: 40–42 mg; Kd: 40–49 mg), and subsequent doses were administered at higher concentrations, as recommended [10]. Approximately half of patients (KRd: 60%–66%; Kd: 53%–55%) received more than two injections in a week at least once during the study. Additionally, approximately half of the patients (KRd: 56%–65%; Kd: 30%–49%) received one injection per week infrequently (Table S1). Carfilzomib dosing patterns in 2018 and 2019 were also compared by treatment group and treatment line (Tables S2 and S3).

Our study showed OS for KRd to be similar to or higher than OS in a previously reported Canadian real‐world study [11], but slightly lower than OS reported in clinical trials (48 months for KRd) [2]. This indicates a potential lack of experience in the treatment management in the real world between 2016 and 2019, and the effectiveness gap between clinical trials and the real world, likely owing to patients in clinical trials having a less severe condition and lower drug exposure than those in the real world [12]. Longer OS and TTNT were observed at 2L/3L than at 4L+, highlighting the importance of using and optimizing KRd in 2L/3L. This is in line with the guideline from the International Myeloma Working Group recommending KRd use in early lines of treatment for patients with RRMM [13].

In our study, OS in patients receiving Kd was lower at 2L than 3L; however, Kd is not generally used in 2L, as demonstrated by the low patient numbers. Therefore, these patients who received Kd in 2L were likely those who had no other treatment options. They were also older at diagnosis, and fewer had prior ASCT than those who received Kd in 3L and 4L+. Despite this, OS was still greater in patients receiving Kd in 2L than in 4L+. OS for carfilzomib monotherapy or doublet therapy was longer (16 months) in the Canadian study than for Kd in this study; however, the Canadian cohort had a greater proportion of patients with prior ASCT and more patients at 2L/3L than in the current study. In a previous study describing the real‐world use of KRd and Kd in patients with RRMM across Europe and Israel [7, 8], patients receiving Kd were older, frailer, and more heavily pretreated than patients receiving KRd; this is consistent with the current study. Differences in patient populations likely explain the shorter OS observed in this study compared with the Canadian cohort, and underline that results for patients receiving Kd and KRd cannot be compared directly.

At the time the study was conducted, drug options were limited; therefore, most patients received bortezomib and lenalidomide prior to KRd and Kd. Prior daratumumab‐based treatment was frequent only among patients receiving KRd or Kd at 4L+. Previous treatment has been reported to affect survival, with shorter OS reported in patients who had not received ASCT and in those who received a lenalidomide‐sparing regimen at 2L [9]. Older patients (≥70 years) and those with multiple comorbidities also had shorter OS [9]. As such, patient characteristics need be taken into account when considering an optimal treatment sequence. Treatment use after KRd and Kd were not analysed in the present study. Previous results showed that patients who received a proteasome inhibitor‐based doublet in 2L typically received pomalidomide in 3L if they continued therapy [9].

Using the nationwide SNDS database allowed analysis of a fully representative population of patients with RRMM. Limitations of our study have been described previously [9]. Notably, these data may no longer reflect current treatment patterns, including the use of newer triplet combinations.

In conclusion, this extensive real‐world analysis of patients with RRMM receiving carfilzomib in the first years after launch in France confirmed that KRd was primarily used in 2L/3L, and use of KRd in these lines is associated with higher OS than when used at later lines. Kd was primarily used in later lines, which reflects the use of this combination in patients with advanced disease (refractory to multiple drugs).

With Kd, KRd, and the more recently approved D‐Kd all available in France, carfilzomib remains an important treatment option for patients with RRMM.

## AUTHOR CONTRIBUTIONS

**Nadia Quignot** and **Gaëlle Désaméricq** designed the study. Data were collected by **Nadia Quignot**. **Nadia Quignot** and **Heng Jiang** analysed the data. All authors contributed to the data interpretation; writing of the manuscript; and approved the final version for publication.

## CONFLICT OF INTEREST STATEMENT

Cyrille Hulin has received a grant and fee from Celgene and Janssen, and fees from Amgen and Takeda. Nadia Quignot and Heng Jiang have received consulting fees from Certara Evidence & Access, Paris, France. Hakima Mechiche and Gaëlle Désaméricq are full‐time employees at Amgen SAS, France, and own stock in Amgen.

## FUNDING INFORMATION

This study was funded by Amgen Europe GmbH.

## ETHICS STATEMENT

The study was performed in accordance with the Declaration of Helsinki and the International Council for Harmonisation guidelines and approved by the French CNIL.

## PATIENT CONSENT STATEMENT

The authors have confirmed patient consent statement is not needed for this submission.

## CLINICAL TRIAL REGISTRATION

The authors have confirmed clinical trial registration is not needed for this submission.

## Supporting information

Supporting Information

## Data Availability

Data were shared by the French national health insurance: “Caisse Nationale d'Assurance Maladie” (CNAM). Access to data in the SNDS is available exclusively to institutions who meet the criteria for access to confidential data, following procurement of consent from the Ethic and Scientific Committee (CESRESS) of the Health Data Hub (HDH) and from the French data protection authority (CNIL).
