# Supplementary material for: Carfilzomib use in patients with relapsed/refractory multiple myeloma in France: A national retrospective cohort study
Source: EJHaem. 2024 Jul 23;5(4):887–91. doi: 10.1002/jha2.946 (PMC11327767; doi:10.1002/jha2.946)
Supplement: Supplementary file 1 — Supporting Information [file JHA2-5-887-s001.docx]

# Supplement

## **Table S1** Dose and frequency of carfilzomib-based regimens.

|  | **KRd (*n* = 993)** | | | **Kd (*n* = 1478)** | | |
| --- | --- | --- | --- | --- | --- | --- |
|  | **2L** | **3L** | **4L+** | **2L** | **3L** | **4L+** |
| Number of patients | 497 | 203 | 293 | 105 | 240 | 1133 |
| Dose (mg/m^2^), first two administrations |  |  |  |  |  |  |
| Median (IQR) | 22 (13–33) | 23 (17–33) | 23 (16–33) | 22 (12–33) | 27 (17–33) | 25 (17–33) |
| Dose (mg/m^2^), subsequent administrations |  |  |  |  |  |  |
| Median (IQR) | 28 (22–33) | 29 (22–33) | 28 (19–33) | 34 (24–48) | 43 (30–57) | 37 (27–54) |
| Number of injections per week |  |  |  |  |  |  |
| Median (IQR)^a^ | 2 (2–3) | 2 (2–3) | 2 (2–3) | 2 (2–4) | 2 (2–3) | 2 (2–3) |
| Patients with always only 1 injection per week (n, %)^a^ | ≤ 10 | ≤ 10 | ≤ 10 | 15 (14.3) | ≤ 10 | 136 (12.0) |
| Patients with change from 2 injections per week to 1 injection per week (n, %)^a^ | 185 (37.2) | 73 (36.0) | 98 (33.4) | 20 (19.0) | 75 (31.3) | 294 (25.9) |
| Patients with change from 1 injection per week to 2 injections per week (n, %)^a^ | 117 (23.5) | 59 (29.1) | 67 (22.9) | 11 (10.5) | 43 (17.9) | 210 (18.5) |
| Patients receiving carfilzomib at home (n, %) | 24 (4.8)​ | ≤ 10 | 14 (4.8)​ | ≤ 10 | ≤ 10 | 66 (5.8)​ |

Patient numbers of ≤ 10 are not specified to comply with data privacy restrictions in France.

^a^This study relies on reimbursed prescription records. The timing of drug reimbursement may not always match the actual administration date exactly; therefore, in some instances, the reported number of injections per week may not precisely reflect the number of administered injections per week.

Abbreviations: 2L, second-line treatment; 3L, third-line treatment; 4L+, fourth-line treatment or later lines; IQR, interquartile range; Kd, carfilzomib and dexamethasone; KRd, carfilzomib, lenalidomide and dexamethasone.

## **Table S2** KRd dosing patterns in 2018 and 2019 (n = 766).

|  | **2018** | | | **2019** | | |
| --- | --- | --- | --- | --- | --- | --- |
|  | **2L** | **3L** | **4L+** | **2L** | **3L** | **4L+** |
| Number of patients | 154 | 70 | 92 | 244 | 86 | 120 |
| Patients with ≥ 2 injections in a week at least once (n, %) | 148 (96.1) | 70 (100) | 85 (92.4) | 230 (94.3) | 84 (97.7) | 113 (94.2) |
| Patients with always only 1 injection per week (%) | ≤ 10 | 0 | ≤ 10 | ≤ 10 | ≤ 10 | ≤ 10 |
| Patients with > 2 injections in a week at least once (n, %) | 112 (72.7) | 48 (68.6) | 52 (56.5) | 140 (57.4) | 60 (69.8) | 84 (70.0) |
| Patients with 1 injection per week infrequently (n, %) | 95 (61.7) | 43 (61.4) | 53 (57.6) | 145 (59.4) | 56 (65.1) | 63 (52.5) |
| Patients with change from 2 injections per week to 1 injection per week (n, %) | 63 (40.9) | 29 (41.4) | 26 (28.3) | 95 (38.9) | 29 (33.7) | 43 (35.8) |
| Patients with change from 1 injection per week to 2 injections per week (n, %) | 32 (20.8) | 14 (20.0) | 27 (29.3) | 50 (20.5) | 27 (31.4) | 20 (16.7) |
| Duration of treatment (months)^a^ |  |  |  |  |  |  |
| Carfilzomib mean (SD) | 9 (5) | 9 (5) | 6 (5) | 7 (3) | 6 (3) | 5 (3) |
| Lenalidomide mean (SD) | 11 (6) | 10 (6) | 7 (5) | 7 (4) | 6 (3) | 5 (3) |

Patient numbers of ≤ 10 are not specified to comply with data privacy restrictions in France.

^a^Treatment duration was computed as ‘last treatment dose date – first treatment dose date + 1)/365.25)*12’. Some patients may have died or been lost to follow-up before the end of their treatment and some patients had only one reimbursed record (15% for all years combined).

Abbreviations: 2L, second-line treatment; 3L, third-line treatment; 4L+, fourth-line treatment or later lines; 5L, fifth-line treatment; KRd, carfilzomib, lenalidomide and dexamethasone; SD, standard deviation; TTNT, time-to-next treatment.

## **Table S3** Kd dosing patterns in 2018 and 2019 (n = 1320).

|  | **2018** | | | **2019** | | |
| --- | --- | --- | --- | --- | --- | --- |
|  | **2L** | **3L** | **4L+** | **2L** | **3L** | **4L+** |
| Number of patients | 25 | 88 | 350 | 65 | 128 | 664 |
| Patients with ≥ 2 injections in a week at least once (n, %) | 24 (96.0) | 82 (93.2) | 321 (91.7) | 55 (84.6) | 120 (93.8) | 576 (86.7) |
| Patients with always only 1 injection per week (%) | ≤ 10 | ≤ 10 | ≤ 10 | ≤ 10 | ≤ 10 | 86 (13.0) |
| Patients with > 2 injections in a week at least once (n, %) | 17 (68.0) | 58 (65.9) | 218 (62.3) | 32 (49.2) | 64 (50.0) | 330 (49.7) |
| Patients with 1 injection per week infrequently (n, %) | ≤ 10 | 41 (46.6) | 155 (44.3) | 17 (26.2) | 66 (51.6) | 289 (43.5) |
| Patients with change from 2 injections per week to 1 injection per week (n, %) | ≤ 10 | 34 (38.6) | 96 (27.4) | ≤ 10 | 34 (26.6) | 159 (23.9) |
| Patients with change from 1 injection per week to 2 injections per week (n, %) | ≤ 10 | ≤ 10 | 59 (16.9) | ≤ 10 | 32 (25.0) | 130 (19.6) |
| Duration of treatment (months)^a^ |  |  |  |  |  |  |
| Carfilzomib mean (SD) | 5 (5) | 7 (5) | 6 (5) | 3 (3) | 4 (3) | 3 (3) |

Patient numbers of ≤ 10 are not specified to comply with data privacy restrictions in France.

^a^Treatment duration was computed as ‘(last treatment dose date – first treatment dose date + 1)/365.25)*12’. Some patients may have died or been lost to follow-up before the end of their treatment and some patients had only one reimbursed record (15% for all years combined).

Abbreviations: 2L, second-line treatment; 3L, third-line treatment; 4L+, fourth-line treatment or later lines; 5L, fifth-line treatment; Kd, carfilzomib and dexamethasone; SD, standard deviation; TTNT, time-to-next treatment.
